# When Carcinoma Meets Sarcoma: A Rare Case of Pancreatic Carcinosarcoma and Review of Literature

**DOI:** 10.1002/ccr3.72416

**Published:** 2026-05-20

**Authors:** Pouria Abedini, Behnam Sanei, Ali Hajihashemi, Maryam Sanei, Alireza Firouzfar

**Affiliations:** ^1^ Student Research Committee, School of Medicine Isfahan University of Medical Sciences Isfahan Iran; ^2^ Department of General Surgery, School of Medicine Isfahan University of Medical Sciences Isfahan Iran; ^3^ Department of Radiology, School of Medicine Isfahan University of Medical Sciences Isfahan Iran

**Keywords:** carcinoma, carcinosarcoma, pancreas, Pancreaticoduodenectomy, sarcoma

## Abstract

Pancreatic carcinosarcoma is an extremely rare and aggressive malignancy with both epithelial and mesenchymal components. This case contributes to the limited global literature, emphasizing the importance of awareness, accurate diagnosis, and further research to improve understanding and management of this lethal tumor.

**Abbreviations**
CBD: common bile duct
CK: pancytokeratin
CT: computed tomography
GI: gastrointestinal
H&E: Hematoxylin and eosin
ICU: intensive care unit
MRCP: magnetic resonance cholangiopancreatography
PCS: Pancreatic carcinosarcoma
WHO: World Health Organization

## 1 Introduction

Pancreatic carcinosarcoma (PCS) is a rare, aggressive pancreatic neoplasm characterized by distinct carcinomatous (epithelial) and sarcomatous (mesenchymal) components. Though carcinosarcoma is predominantly observed in the genitourinary tract, its occurrence in the pancreas remains exceptionally rare, with limited reports documented to date [1]. Accurate diagnosis depends critically on the clear identification of biphasic pathology featuring both epithelial and mesenchymal components [2]. Three theories—the transformation, collision, and combination theories—have been proposed to explain the origin of carcinosarcomas. The transformation theory suggests that carcinoma undergoes metaplasia to sarcoma; the collision theory posits independent simultaneous malignant growths merging, and the combination theory hypothesizes a single multipotent stem cell origin differentiating into dual components [3].

The World Health Organization (WHO) classifies PCS as an undifferentiated carcinoma of the pancreas, underscoring the tumor's high aggressiveness and poor prognosis, with a median survival of about 6 months [4, 5]. Currently, no definitive therapy exists for PCS, making surgical resection the cornerstone of treatment, potentially extending patient survival when performed at an early stage [4]. Considering the diagnostic and therapeutic challenges, this case report significantly contributes to the literature as the first reported case of PCS from Iran.

## 2 Case History

### 2.1 Patient Information

A 73‐year‐old male with a history of diabetes mellitus, rheumatoid arthritis, and hypertension presented to the emergency department of our university‐affiliated hospital with massive hematemesis and persistent generalized abdominal pain. He reported a gradual decline in appetite and progressive weight loss over the past few months. On physical examination, he appeared pale and tachycardic, with a heart rate of 118 beats per minute and a blood pressure of 100/65 mmHg. Despite the presence of significant gastrointestinal (GI) bleeding, no tenderness, guarding, or peritoneal signs were noted on abdominal examination.

### 2.2 Past Medical History

**FIGURE 1:**
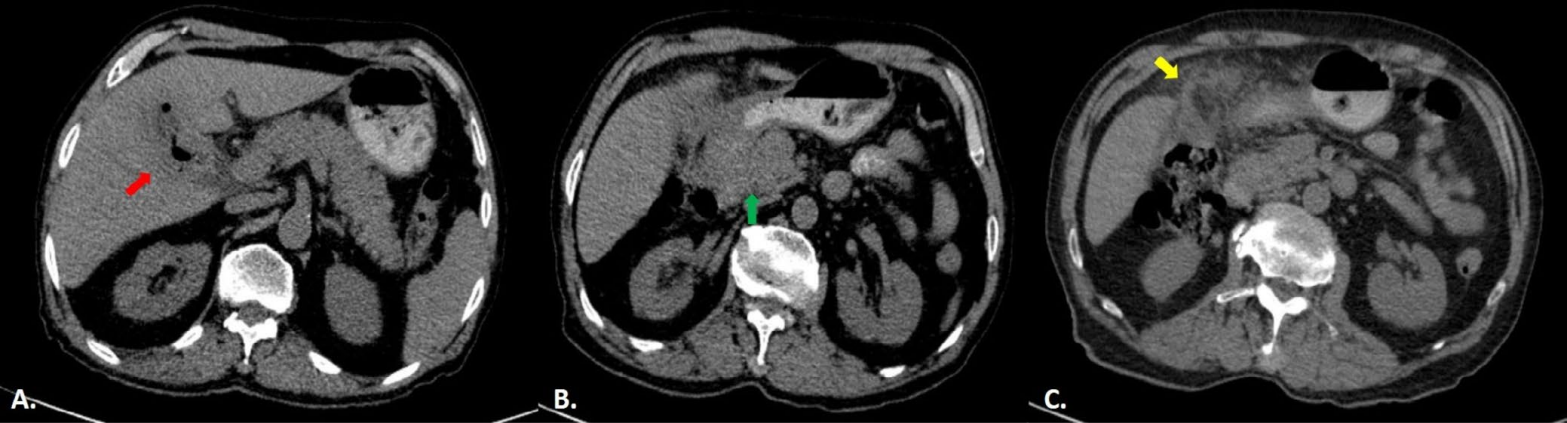
Abdominopelvic CT scan without IV contrast. (A) Gallbladder has an indistinct margin and contains intraluminal air foci (red arrow), suggestive of a cholecystoduodenal fistula. (B) Hypodense ill‐defined mass in pancreatic head with invasion into the adjacent duodenum (green arrow). (C) Fat stranding and soft tissue density in mesentery of hepatic flexure of colon (yellow arrow), suggestive of mesenteric invasion.

**FIGURE 2:**
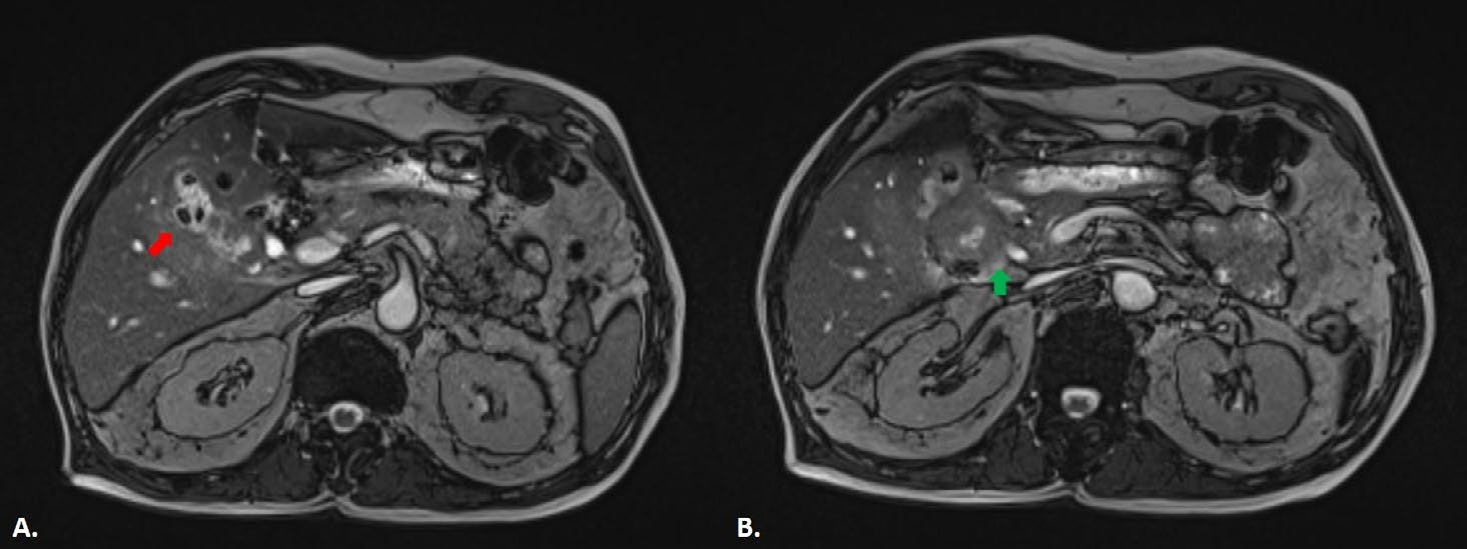
Balanced turbo field echo sequence of MRCP. (A) Wall thickening and irregularity of the gallbladder associated with low signal foci (intraluminal air) (red arrow). (B) Iso‐signal mass in the pancreatic head with invasion to the duodenum and hilum of the liver (green arrow).

One month prior to admission, the patient had undergone both abdominopelvic computed tomography (CT) without IV contrast and magnetic resonance cholangiopancreatography (MRCP) due to ongoing gastrointestinal symptoms. The CT scan demonstrated gallbladder wall thickening with indistinct margins containing intraluminal air foci suggestive of a cholecystoduodenal fistula. Additionally, a hypodense ill‐defined mass in the pancreatic head with invasion into the duodenum and hepatic hilum was seen, associated with soft tissue stranding in the mesentery of the hepatic flexure of the colon, consistent with mesenteric invasion (Figure 1). MRCP showed mild intrahepatic biliary ectasia, common bile duct (CBD) dilation with a maximum diameter of 10 mm with a 2 mm distal CBD stone, gallbladder wall thickening (6 mm), and an iso‐signal mass in the pancreatic head extending toward the duodenum and hepatic hilum (Figure 2). These findings suggest a complex inflammatory or neoplastic process involving the pancreatic head, the gallbladder, and the duodenum. In addition to imaging studies, the patient had undergone endoscopy and colonoscopy 1 month prior to surgery. Upper endoscopy revealed diffuse erythematous gastric mucosa with scattered erosions in the body and antrum, necessitating multiple biopsies. The duodenum exhibited a deep, 20 mm ulcer with a clean base, which appeared suspicious for malignancy or represented an orifice to a fistula. Colonoscopy findings included five sessile polyps ranging from 2 to 5 mm in size in the proximal transverse and ascending colon, all of which were removed. Histopathological examination of the duodenal biopsy demonstrated spindle‐shaped cells with epithelioid features and mitotic activity without atypia, suggestive of a mesenchymal neoplasm. Gastric antral biopsy revealed chronic gastritis without *Helicobacter pylori* . The histological evaluation of the colonic polyps confirmed tubular adenomas.

## 3 Methods

### 3.1 Initial Laboratory and Imaging Findings

Initial laboratory tests revealed a white blood cell count of 24.8 × 10^3^/μL, hemoglobin of 9.5 g/dL, and a platelet count of 528 × 10^3^/μL. Liver function tests showed AST and ALT levels of 19 and 12 U/L, respectively. According to the severity of GI bleeding, resuscitation, including blood transfusion, was performed. The patient subsequently underwent upper gastrointestinal endoscopy, which revealed a giant duodenal ulcer with a large visible vessel.

### 3.2 Surgical Intervention

Given the clinical deterioration and concern for a malignant process, a multidisciplinary team—including specialists in surgery, internal medicine, cardiology, and anesthesiology—assessed the case. Because of the endoscopy report and recurrent GI bleeding, the consensus was performing a surgical intervention. Intraoperative findings confirmed an invasive pancreatic tumor with extensive local involvement. The surgery included a Whipple procedure, partial hepatectomy of segments 4 and 5 (due to macroscopic involvement), right hemicolectomy (due to mesenteric involvement of the right colon), and lymph node dissection, so complete tumor resection was achieved. There was no evidence of ascites or peritoneal seeding. After performing duct‐to‐mucosa pancreaticojejunostomy, three separate hepatojejunostomies were performed to reconstruct biliary continuity by anastomosing the left, mid, and right hepatic ducts to the jejunal limb.

## 4 Conclusions and Results

### 4.1 Postoperative Course and Follow‐Up

The patient's overall health was improved gradually during intensive care unit (ICU) management. Specialists in nutrition and clinical pharmacology managed the patient. There was no evidence of anastomosis leakage during hospitalization. Two months later, he was admitted with severe inappetence, lower limb edema, chills, constipation for 4 days, and urinary retention. On admission, he was hypotensive, tachycardic, and exhibited leukocytosis. The resuscitation process included placement of a central venous line, total parenteral nutrition, and ICU care. However, despite aggressive supportive measures, he developed cardiorespiratory arrest and died because of disease progression.

### 4.2 Pathological Examination

The resected specimen consisted of the pancreas, duodenum, right colon, segments 4 and 5 of the liver, and the liver hilum lymph nodes. On gross examination, the pancreas measured 7.0 × 5.0 × 5.0 cm. A necrotic, encapsulated mass measuring 4.0 cm in its greatest dimension was identified in the pancreatic head. All surgical margins were free of malignancy. The final staging was determined to be T2N0M1, given the absence of regional lymph node involvement but the presence of sarcomatous metastatic spread to the liver.

**FIGURE 3:**
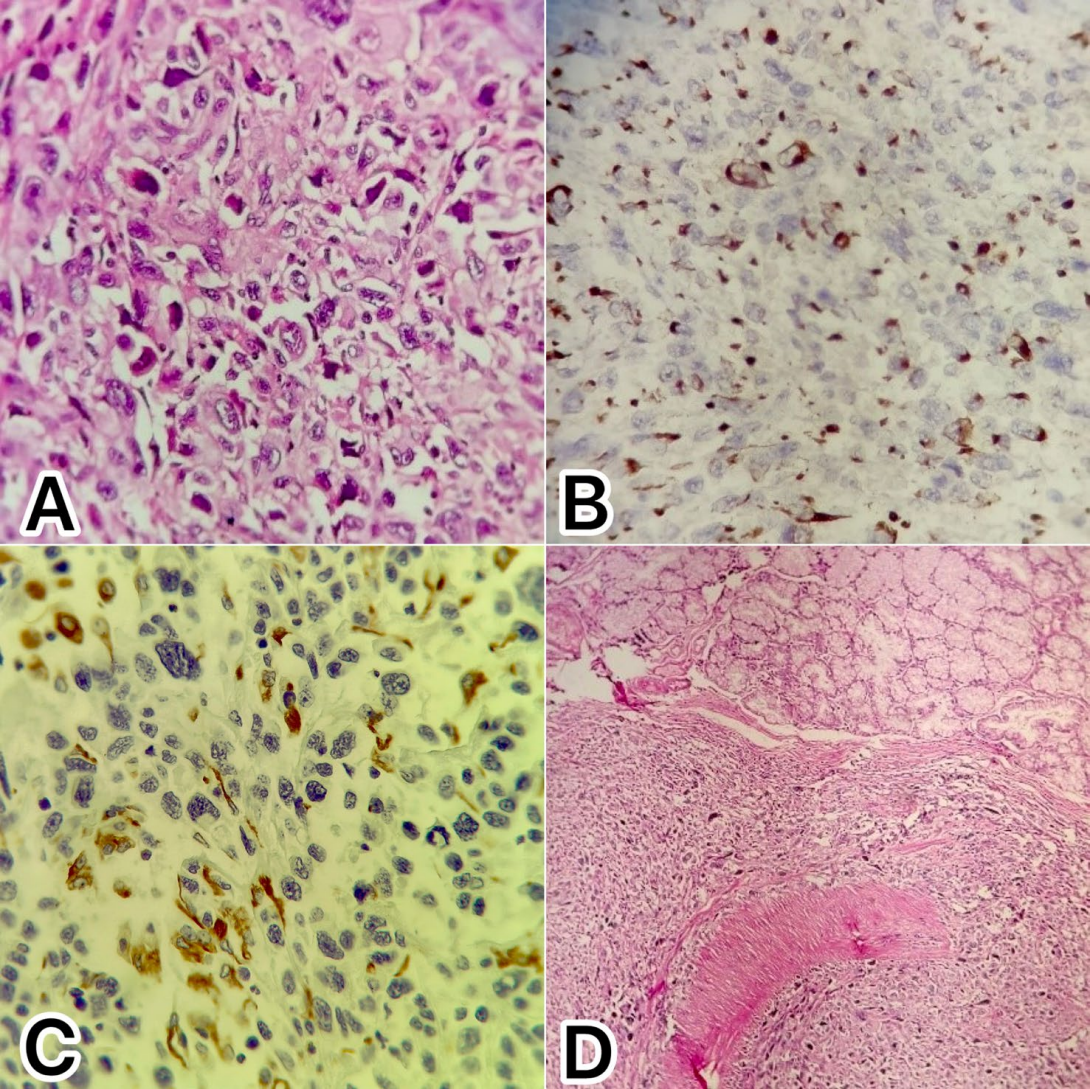
(A) Hematoxylin and eosin (H&E) staining of the pancreatic head showing densely packed spindle cells with pleomorphic nuclei and brisk mitotic activity, indicative of the sarcomatous component. Scattered epithelial cells are also present and highlighted by cytokeratin immunostaining in Figure 3B (400×). (B) Immunohistochemical staining for pancytokeratin (CK) demonstrating strong cytoplasmic positivity in scattered epithelial cells, confirming the carcinomatous (ductal adenocarcinoma) component corresponding to the area shown in Figure 3A (400×). (C) Immunohistochemical staining for vimentin demonstrating diffuse positivity within the spindle cell component, consistent with mesenchymal differentiation (×400). (D) H&E staining of the pancreatic head showing invasion of the tumor into the duodenal wall (100×).

Histopathological examination revealed a biphasic malignant neoplasm composed of distinct epithelial and mesenchymal components. Hematoxylin and eosin (H&E) staining demonstrated fascicles of pleomorphic spindle cells with hyperchromatic, irregular nuclei and frequent mitotic figures, consistent with the sarcomatous component (Figure 3A). Immunohistochemical staining for pancytokeratin (CK) showed strong cytoplasmic positivity in the epithelial component, supporting the presence of ductal adenocarcinoma (Figure 3B). Immunohistochemical staining for vimentin demonstrated diffuse cytoplasmic positivity within the spindle cell component, consistent with mesenchymal differentiation (Figure 3C). These findings support biphasic differentiation in keeping with pancreatic carcinosarcoma. Tumor infiltration into the duodenal wall was also confirmed histologically (Figure 3D).

A total of 21 lymph nodes were examined, all of which were negative for metastatic involvement. No lymphovascular invasion was observed. The positivity of immunohistochemical staining for CK and negativity for DOG‐1, S100, CD34, and Desmin confirmed the final diagnosis of pancreatic carcinosarcoma.

## 5 Discussion

Pancreatic cancer is the 7th cause of cancer mortality with approximately 6% of 5‐year survival, and is found to carry a poor prognosis [6]. Based on the latest edition of the WHO classification for pancreatic cancers, undifferentiated carcinoma of the pancreas, as a subtype of pancreatic ductal adenocarcinoma, is divided into two main groups, including undifferentiated carcinoma and undifferentiated carcinoma with osteoclast‐like giant cells. The first group is also classified into three subcategories: anaplastic undifferentiated carcinoma, sarcomatoid carcinoma, and carcinosarcoma [5, 7, 8]. After conducting a comprehensive literature search in PubMed and excluding unrelated articles, 51 cases were analyzed and summarized in Table 1. Our review identified a mean patient age of approximately 60.8 years, with a slight male predominance (51%), and 64.1% of cases occurring in the pancreatic head. The clinical and pathological features observed in our case closely match these findings.

**TABLE 1:** Reported Cases of Pancreatic Carcinosarcoma from 1951 to 2025: Clinical, Pathological, and Therapeutic Characteristics Ordered by Year of Publication.

References	Age/sex	Symptom	Location in the pancreas	Size	Biopsy method & diagnosis	Neoplastic invasion	Carcinomatous component	Sarcomatous component	Type of surgery	Chemotherapy	Survival (month)	Cause of death
Van Damme and Snoeks [9]	—		B		—				—		—	—
Millis et al. [10]	—	—	H	—	—	Duodenum	Adenocarcinoma	Leiomyosarcoma	PD		—	—
Watanabe et al. [11]	76/M	—	H	5 cm	—	—	Ductal adenocarcinoma	Mixed osteoclastic/pleomorphic‐type giant cell tumor	—		3	Cholangitis with multiple liver abscesses
Darvishian et al. [12]	74/M	Deep venous thrombosis of lower limb	H	4.0 × 3.0 × 3.0 cm	ERCP with brushings: adenocarcinoma	Peripancreatic adipose tissue and duodenal wall	Ductal adenocarcinoma	Malignant fibrous histiocytoma	PD		> 4	—
Yamazaki [13]	90/M	—	—		—	—	Adenocarcinoma	Small round cells: Undifferentiated short cells of spindle shaped	—		> 1	Cachexia due to generalized tumor extension
Barkatullah et al. [14]	67/F	Abdominal Pain	H	2.5 × 2.5 × 2.0 cm	EUS FNA: poorly differentiated carcinoma with multinucleated giant cells	No	Adenocarcinoma	Spindle cells sarcoma and osteoclast giant cell‐rich spindle cell proliferation	PD		8	Liver metastasis
Chmiel et al. [15]	47/M	Jaundice	H	—	—	—	Adenocarcinoma	Leyosarcoma	PD		—	—
Bloomston et al. [16]	67/F	Nausea, vomiting, and jaundice	H	4.0 × 4.0 × 3.0 cm	—	Duodenum	Mucinous cystadenocarcinoma	Sarcomatous stroma	PD		4	Peritoneum and liver metastasis
Nakano et al. [17]	82/F	Jaundice	H	18 × 11 × 10 cm	—	Transverse mesocolon	Adenocarcinoma	Spindle cells	PD, partial resection of colon		0.5	Postoperative complications
Gelos et al. [18]	61/F	Anemia and physical examination	H	7.0 × 6.0 × 3.5 cm	—	Peripancreatic adipose tissue	Carcinoma	Spindle cells	PD	Gemcitabine	11	Tumor recurrence
Okamura et al. [19]	64/F	Physical examination	T	3.5 × 2.1 × 1.4 cm	—	No	Intraductal papillary mucinous carcinoma	Osteosarcoma	DP		> 12	—
Shen et al. [20]	71/F	Nausea, vomiting, and abdominal pain	H	5 × 4 × 4 cm	—	Peripancreatic adipose tissue and duodenal wall	Ductal adenocarcinoma	Malignant fibrous histiocytoma	PD, partial hepatic resection, gastric wedge resection		2	Liver metastasis and tumor recurrence in the tail of pancreas
Kim et al. [21]	48/M	Physical examination	T	3.5 × 2.5 × 1.5 cm	—	Peripancreatic adipose tissue	Mucinous cystadenocarcinoma	Pleomorphic spindle cells	DP, segmental resection of colon	3 cycles of gemcitabine	4	Peritoneum and liver metastasis
Zhu et al. [22]	53/F	Abdominal pain and jaundice	H	5.0 × 4.0 × 3.0 cm	—	Duodenum	Ductal adenocarcinoma	Pleomorphic spindle cells	PD	5 cycles of gemcitabine, doxorubicin, cisplatin	> 20	—
Oymaci et al. [23]	66/M	Abdominal pain jaundice	H	3.5 × 2.0 × 1.5 cm	ERCP with brushings: adenocarcinoma	Peripancreatic adipose tissue and duodenal wall	Ductal adenocarcinoma	Malignant fibrous histiocytoma	PD		0.66	Postoperative complications
Shi et al. [24]	74/F	Abdominal Pain	T	5.0 × 4.0 × 2.0 cm	—	—	Ductal adenocarcinoma	Spindle cells	DP		—	—
Lee et al. [25]	24/F	Abdominal Pain	T	4.7 × 3.5 cm	—	Peripancreatic soft tissue	Solid and pseudopapillary tumor	Spindle cells	DP		—	—
Bai et al. – case1 [3]	71/M	Overall mentioned symptoms: Abdominal pain–Jaundice–Nausea and vomiting–Anemia–Anorexia—Weight loss–Incidental mass discovery	H	Approximately 5 cm	Histopathology & IHC, Pancreatic carcinosarcoma	Overall mentioned: Invasion into the surrounding pancreatic tissue and peripancreatic fat	Ductal adenocarcinoma	Malignant fibrous histiocytoma/undifferentiated pleomorphic sarcoma + osteosarcoma	PDE + SE + POC	Gemcitabine + Floxuridine	11	Tumor progression
Bai et al. – case2 [3]	49/M	Overall mentioned symptoms: Abdominal pain–Jaundice–Nausea and vomiting–Anemia–Anorexia—Weight loss–Incidental mass discovery	H	5 cm	Histopathology & IHC	Overall mentioned: Invasion into the surrounding pancreatic tissue and peripancreatic fat	Ductal adenocarcinoma	Osteosarcoma + MFH/UPS	PDE + POC	Gemcitabine + Adriamycin + Cisplatin	39	
Bai et al. – case3 [3]	74/M	Overall mentioned symptoms: Abdominal pain–Jaundice–Nausea and vomiting–Anemia–Anorexia—Weight loss–Incidental mass discovery	H	8 cm	Histopathology & IHC	Overall mentioned: Invasion into the surrounding pancreatic tissue and peripancreatic fat	Ductal adenocarcinoma	Pleomorphic spindle‐cell sarcoma	PDE	None	10	Tumor progression
Bai et al. – case4 [3]	38/M	Overall mentioned symptoms: Abdominal pain–Jaundice–Nausea and vomiting–Anemia–Anorexia—Weight loss–Incidental mass discovery	T	16 cm	Histopathology & IHC	Overall mentioned: Invasion into the surrounding pancreatic tissue and peripancreatic fat	Mucinous cystadenocarcinoma	Pleomorphic spindle‐cell sarcoma	DPE + SE + GKRS + POC	Gemcitabine + Cisplatin + Adriamycin + Paclitaxel + GKRS (gamma knife)	26	Alive with recurrence
Bai et al. – case5 [3]	67/M	Overall mentioned symptoms: Abdominal pain–Jaundice–Nausea and vomiting–Anemia–Anorexia—Weight loss–Incidental mass discovery	H	3.5 cm	Histopathology & IHC	Overall mentioned: Invasion into the surrounding pancreatic tissue and peripancreatic fat	Ductal adenocarcinoma	Embryonalrhabdomyosarcoma	PDE + POC	Gemcitabine + Cisplatin + Adriamycin	47	Tumor recurrence
Bai et al. – case6 [3]	60/F	Overall mentioned symptoms: Abdominal pain–Jaundice–Nausea and vomiting–Anemia–Anorexia—Weight loss–Incidental mass discovery	B/T	7.5 cm	Histopathology & IHC	Overall mentioned: Invasion into the surrounding pancreatic tissue and peripancreatic fat	Mucinous cystadenocarcinoma	Malignant fibrous histiocytoma/undifferentiated pleomorphic sarcoma	PDE	None	15	Tumor progression
Bai et al. – case7 [3]	75/F	Overall mentioned symptoms: Abdominal pain–Jaundice–Nausea and vomiting–Anemia–Anorexia—Weight loss–Incidental mass discovery	H	4.5 cm	Histopathology & IHC	Overall mentioned: Invasion into the surrounding pancreatic tissue and peripancreatic fat	Ductal adenocarcinoma	Pleomorphic spindle‐cell sarcoma	PDE + TCMHT	TCMHT	29	
Bai et al. – case8 [3]	59/M	Overall mentioned symptoms: Abdominal pain–Jaundice–Nausea and vomiting–Anemia–Anorexia—Weight loss–Incidental mass discovery	B/T	7.5 cm	Histopathology & IHC	Overall mentioned: Invasion into the surrounding pancreatic tissue and peripancreatic fat	Ductal adenocarcinoma	Malignant fibrous histiocytoma/undifferentiated pleomorphic sarcoma	PDE + SE + POC	Gemcitabine + Adriamycin + Cisplatin + Paclitaxel	17	
Salibay et al. [26]	49/F	Abdominal and back pain, weight loss	B & T	—	Biopsy during hysterectomy: high‐grade spindle cell sarcoma & moderately differentiated adenocarcinoma	Superior mesenteric artery, hepatocholedochal lymph node	Moderately differentiated adenocarcinoma	High‐grade spindle cell sarcoma	Unresectable tumor due to involvement of the superior mesenteric artery	Gemcitabine & docetaxel, followed by ifosfamide & doxorubicin	10	Progression of disease
Mszyco et al. [27]	85/M	Abdominal pain and weight loss	H	12 cm	EUS FNA: atypical spindle & myxoid type sarcoma	—	Adenocarcinoma	Spindle cells	PD		2.5	—
Ruess et al. [28]	73/F	Abdominal pain and physical examination	H	6.5 × 4.5 × 3 cm	—	Peripancreatic adipose tissue	Ductal adenocarcinoma	Spindle cells	PD		4	—
Li et al. [29]	60/M	Steatorrhea, weight loss and physical examination	H	10 × 9 × 9 cm	—	Muscular layer of the duodenum	Adenocarcinoma and squamous carcinoma cells	Cartilaginous and osteal differentiation	Total pancreatectomy		> 7	—
Jia et al. [30]	44/F	Abdominal pain and jaundice	H	3 cm	—	fat tissues surrounding the head of the pancreas and lymph node metastasis	Adenocarcinoma	Osteosarcoma	PD	8 cycles of gemcitabine & raltitrexed	> 31	—
Still et al. [31]	59/F	Abdominal pain, nausea, and vomiting	H	2.5 cm	EUS FNA: adenocarcinoma	Duodenum, main pancreatic duct, and intrapancreatic bile duct	Adenocarcinoma	High‐grade spindle cell with focal chondrosarcoma and myogenic differentiation	PD	6 cycles of modified FOLFIRINOX and 1 cycle of gemcitabine & paclitaxel	10	Liver metastasis
Zhou et al. [32]	44/F	Abdominal pain and jaundice	H	3.0 × 2.0 × 1.6 cm	—	Peripancreatic adipose tissue and the common bile duct wall	Ductal adenocarcinoma	Pleomorphic spindle cells	PD		> 48	—
Liu et al. [33]	66/M	Abdominal pain, nausea, jaundice	H	5.0 × 4.0 × 4.0 cm	—	—	Ductal adenocarcinoma	Undifferentiated (pleomorphic tumor cells)	Cholecystectomy with bile duct‐jejunum Roux‐en‐Y anastomosis, radioactive seed implantation	Radioactive seed implantation at surgery	> 12	—
Quinn et al. [34]	42/F	Abdominal pain with radiation to back, nausea, bloating, anorexia, and diarrhea	B & T	10 cm	Transgastric FNA: adenocarcinoma	Spleen, colon	Mixed mucinous adenocarcinoma	Spindle cells	Subtotal pancreatectomy, left partial adrenalectomy, left hemicolectomy, and splenectomy	9 cycles of gemcitabine & paclitaxel, 11 cycles of FOLFOX	> 16	—
Li et al. [35]	60/M	Abdominal pain	T	7.5 cm		Vascular invasion		undifferentiated tumor cells with spindle morphology	Total pancreatectomy		2	—
Li et al. [35]	66/M	Painless jaundice	H	4.0 cm		Vascular invasion		undifferentiated tumor cells with spindle morphology	PD	Gemcitabine plus Nab‐paclitaxel	11	—
Li et al. [35]	69/M	Incidental finding	H	2.5 cm		—		undifferentiated tumor cells with spindle morphology	PD	mFOLFIRINOX	19	—
Li et al. [35]	56/F	Right upper quadrant pain, jaundice	H	10.0 cm		Vascular invasion		undifferentiated tumor cells with spindle morphology	Total pancreatectomy		39	—
Li et al. [35]	51/M	Epigastric pain, jaundice	H	4.5 cm		Vascular invasion		undifferentiated tumor cells with spindle morphology	PD	Gemcitabine	17	—
Li et al. [35]	48/F	Epigastric pain	T	8.0 cm		Vascular invasion		undifferentiated tumor cells with spindle morphology	Total pancreatectomy		—	—
Li et al. [35]	67/F	Epigastric pain	H	6.4 cm		—		undifferentiated tumor cells with spindle morphology	PD		4	—
Li et al. [35]	59/M	Abdominal pain	H	5.3 cm		—		undifferentiated tumor cells with spindle morphology	PD		—	—
Li et al. [35]	49/M	Abdominal pain	B	8.0 cm		Vascular invasion		undifferentiated tumor cells with spindle morphology	DP		—	—
Khan et al. [36]	68/M	Abdominal pain, jaundice, nausea, anorexia, loose stools	H	8.2 × 7.9 × 7.2 cm	EUS FNA: High‐grade dysplasia	—	Ductal adenocarcinoma	Spindle cells	PD		3	—
Cheng et al. [37]	73/F	Abdominal pain and jaundice	H	3.0 × 2.0 × 1.6 cm	—	Distal common bile duct and peripancreatic adipose tissue	Ductal adenocarcinoma	Malignant spindle‐shaped and pleomorphic cells (osteosarcoma)	PD		> 48	—
Lalonde et al. [2]	52/F	Diarrhea	H	3.6 cm	EUS FNA: adenocarcinoma	Common bile duct, duodenum, and peripancreatic soft tissue	Undifferentiated carcinoma with osteoclast‐like giant cells	Prominent therapy‐related changes and patchy malignant osteoid/chondroid matrix depositions	PD	4 cycles of FOLFIRINOX, radiation with concurrent capecitabine	> 15	—
Lee et al. [38]	65/F	Abdominal pain and weight loss	H	1.7 × 1.5 × 0.9 cm	EUS FNA: carcinosarcoma	—	Adenocarcinoma	Single cells with severe atypia	PD	11 cycles of FOLFIRINOX, pembrolizumab and	> 7	—
Osama et al. [39]	62/M	Abdominal pain, nausea, vomiting, diarrhea, and weight loss	T	3.0 × 2.8 cm	EUS FNA: anaplastic (pleomorphic) giant cell carcinoma	—	Adenocarcinoma	Spindle cells	—	Chemotherapy and palliative care	Few months after diagnosis	Massive metastasis
Zhou et al. [40]	56/M	Acute abdominal pain due to tumor rupture	—	—	Histopathological examination postsurgery	Tumor rupture into the peritoneal cavity	Ductal adenocarcinoma	High‐grade spindle cell sarcoma	Radical surgical resection	Adjuvant chemotherapy (specific regimen not specified)	20	—
Gilani et al. [41]	60/F	Abdominal pain and jaundice	H	2.0 × 2.2 × 2.0 cm	EUS FNB: carcinoma	Lymphovascular and perineural invasion	Glandular differentiation	Sarcomatoid differentiation	PD	Gemcitabin, capecitabine and FOLFIRINOX	36	—
Yünlüel et al. [42]	40/M	Dyspeptic symptoms and hematemesis	T	2.5 × 2.0 × 1.2 cm	Endoscopy: undifferentiated carcinoma	Gastric wall and left surrenal gland			Subtotal pancreatectomy, splenectomy, and gastric wedge resection	16 courses of radiotherapy (RT) and four cycles of docetaxel + cisplatin	1.53	Confusion and a worsening general condition

*Note:* Data were extracted from published case reports and series. Some reports lacked full information on all parameters. Cases were ordered based on the earliest identifiable publication year. Transitional zones and biphasic morphology were key criteria in distinguishing carcinosarcoma from sarcomatoid carcinoma. Only confirmed carcinosarcoma cases were included in this summary.

Abbreviations: B, Body of pancreas; DP, Distal pancreatectomy; ERCP, Endoscopic retrograde cholangiopancreatography; IHC, Immunohistochemistry; EUS FNA, Endoscopic ultrasound‐guided fine needle aspiration; GKRS, Gamma Knife Radiosurgery; H, Head of pancreas; MFH, Malignant Fibrous Histiocytoma; PD, Pancreaticoduodenectomy (Whipple procedure); PDE, Pancreaticoduodenectomy with extended resection; POC, Postoperative chemotherapy; RT, Radiotherapy; SE, Splenectomy; T, Tail of pancreas; TCMHT, Traditional Chinese Medicine Hyperthermia Therapy; UPS, Undifferentiated Pleomorphic Sarcoma.

A critical diagnostic challenge lies in differentiating PCS from sarcomatoid carcinoma. Both are undifferentiated pancreatic carcinomas with similar histological presentations, including epithelial and mesenchymal components. However, carcinosarcoma is strictly defined by a distinct biphasic morphology without a transitional zone, in contrast to sarcomatoid carcinoma, which typically exhibits a transitional zone between epithelial and mesenchymal elements and can present either monophasically or biphasically [24, 43]. The absence of such transitional zones in our patient strongly supports the diagnosis of pancreatic carcinosarcoma. Accurate distinction between these entities may have implications for understanding tumor pathogenesis, biological behavior, and potential prognostic assessment. Molecular profiling using next‐generation sequencing has been proposed in selected cases to further investigate tumor biology, including the potential clonal relationship between epithelial and mesenchymal components in biphasic malignancies [44, 45]. However, such analysis was not performed in the present case. The diagnosis was established based on distinct histomorphological features and immunohistochemical evidence of biphasic differentiation, which remain the cornerstone for the diagnosis of PCS in routine clinical practice. Additionally, a gross photograph of the resected tumor was not available, which may limit comprehensive clinicopathological correlation.

The aggressive clinical course and poor prognosis of PCS remain consistent across the literature, with median survival rates rarely exceeding 6 months [4]. As seen in our patient, the rapid postoperative progression leading to death underscores the severity of PCS. Despite aggressive surgical intervention, metastatic spread, especially to the liver and peritoneum, frequently leads to poor outcomes [14, 16, 20, 21, 31].

Treatment strategies currently remain limited to surgical resection and, in some cases, conventional chemotherapy regimens such as gemcitabine and FOLFIRINOX [2, 3, 18, 21, 22, 26, 30, 31, 34, 38, 39, 40, 41, 42]. Recent literature emphasizes the urgency for novel therapeutic approaches, including targeted molecular therapy and immunotherapy, which hold promise for treating other aggressive malignancies [46, 47, 48]. Despite the consideration and application of various immunotherapy pathways, such as including checkpoint inhibitors, CD40 agonists, vaccines, bi‐specific antibodies, and chimeric antigen receptor T‐cell therapies, pancreatic cancer continues to have a high mortality rate [46].

As this is the first reported case of PCS in Iran, our findings highlight the need for increased clinical awareness, earlier diagnosis through better imaging modalities, and an improved understanding of this tumor's molecular biology. Exploring genetic and molecular profiles in PCS could reveal actionable targets for future treatments, potentially improving prognosis in future cases [3].

In conclusion, our case significantly contributes to the global understanding of PCS by presenting clear pathological differentiation from sarcomatoid carcinoma, underscoring its aggressive clinical behavior, and reinforcing the critical need for novel therapeutic strategies to improve patient outcomes.

## Author Contributions

**Pouria Abedini:** data curation, validation, writing – original draft. **Behnam Sanei:** project administration, supervision, validation, writing – review and editing. **Ali Hajihashemi:** investigation, supervision, visualization, writing – review and editing. **Maryam Sanei:** writing – original draft, writing – review and editing. **Alireza Firouzfar:** data curation, methodology, writing – review and editing.

## Funding

The authors have nothing to report.

## Ethics Statement

This study obtained ethical approval based on the code of IR.ARI.MUI.REC.1404.056. and all processes done in the study involving human attendance were in accordance with the ethical standards of the Research Ethics Committees of the School of Medicine, Isfahan University of Medical Sciences, Isfahan, Iran and with the Declaration of Helsinki and its later amendments.

## Consent

Written informed consent was obtained from the patient for publication of this case report and any accompanying images.

## Conflicts of Interest

The authors declare no conflicts of interest.

## Data Availability

The datasets used during the current study are available from the corresponding author on reasonable request.
